# Supplementary material for: An unscheduled switch to endocycles induces a reversible senescent arrest that impairs growth of the Drosophila wing disc
Source: PLoS Genet. 2024 Sep 3;20(9):e1011387. doi: 10.1371/journal.pgen.1011387 (PMC11398662; doi:10.1371/journal.pgen.1011387)
Supplement: Attachment — Submitted filename: Huang et al Response to Review.docx [file pgen.1011387.s012.docx]

**Huang et al. Response to Reviews**

We would like to thank the reviewers for their suggestions that have helped us to improve the manuscript. We have performed additional experiments suggested by the reviewers, which has strengthened the original conclusions. We have uploaded both a clean version of text and one in which significant changes are in blue font. Below is a list of new figure panels followed by our itemized response to review.

New Figure Panels

Figure 3C, M: Quantification of DCP-1 labeling

Figure 3G-J: Evaluation of effect of p35 on iEC disc undergrowth at 96 hr.

Figure 7 D-E': Labeling of cell cortex and multipolar mitotic spindle in iECs returning to division.

Figure 7 F-I': Labeling for SA-beta-gal in longer time points after return to division.

Figure 7 J-L''': G-Trace lineage analysis of daughter cells of iEC after return to division.

Figure 8 C-D: Quantification of iEC-induced compensatory proliferation.

Figure 8 E-F": Labeling of anterior (Ci) and posterior (En) compartments shows that iECs maintain posterior fate while anterior cells are induced to partially regenerate lost tissue.

Figure S4: Alternative induction of iEC with Fzr results in a senescent arrest.

Figure S5E-H''': H2aV repair foci labeling in discs at different times after IR - IR induces DNA damage in all cells, which are lost and replaced in the diploid anterior but not polyploid posterior.

Figure S6 A-D: Overexpression of activated Jun kinase kinase (hep^CA^) in salivary glands inhibits developmental endocycles.

**Reviewer #1:**

Overall, this is a timely, rigorous, and intriguing study!

**Response:** Thank you for your encouraging words.

Major Concern:
Validate that the senescent-like state is not specific to the overexpression of Rux by inducing polyploidy via another genetic perturbation (i.e. Fizzy-related, CycE, or E2F1 RNAi). Then repeating the wing disc staining with MMP1 and beta-gal (Figure 5) would be sufficient to address this point.

**Response:** We have induced endocycles by overexpressing Fizzy-related (Cdh1), which also resulted in a growth arrest and expression of the senescent marker SA-beta-gal (Fig.S4). This result indicates that the senescent arrest is a property of unscheduled endocycles and not specific to Rux overexpression.

Minor Concern:
None. A very well written manuscript which nicely puts the significance of the study in the context of the field.

**Reviewer #2:**

The experiments in support of these observations are very well performed and nicely described. The major new advance is the description of how JNK signaling controls the behavior of iEC and the consequent effects on tissue growth, and this information should be helpful when thinking about pathological iEC as has been observed in several cancers. The comments below are intended to help the authors improve the manuscript.

1. Many readers, particular non-fly readers, likely won’t know what rux is or why it induces iEC. I suggest adding a sentence (e.g. page 5) and a citation(s) indicating that rux a cyclin A/cdk inhibitor and that loss of the activity of this kinase is a common way of inducing endoreplication.

**Response:** In the original submission, we had stated that Rux is a Cyclin A inhibitor and cited the previous studies that used it to generate iEC. As suggested, we have added a clause to stress that it is a common way to induce unscheduled endocycles. (pg. 6, para 3).

2. Most of the experiments are well quantified, but several are not and it’s unclear why not. e.g. Figure 3A-G, Figure 7, and some others. Are some stainings not readily quantifiable? Probably should be consistent throughout the manuscript.

**Response** We had not included quantification for some images that were representative of the extreme difference from controls. To increase rigor, however, we have now quantified Dcp-1 labeling (Fig. 3C,M) and EdU labeling during compensatory proliferation (Fig 8C,D).

3. In Figure S4A there is no control showing how much damage was induced by the IR, making the negative result more difficult to interpret. In the subsequent panels these data are shown. Can we assume that 4000 rads always uniformly induces the same amount of damage in every disc?...

**Response:** The experiment in S4A was performed by collecting samples 48 hours post IR. We have now labeled disc 4 hours post IR with H2aV, which demonstrates the known ability of gamma rays to induce high levels of DNA damage in all cells (Fig S5G-H"').

...And why is there Dcp1 staining in the posterior half of the control disc AND the anterior half of the experimental disc in panels S4E’’’ and S4F’’’?...

**Response:** Both samples have been irradiated, which induces DNA damage and sporadic apoptosis. In the control discs, both anterior and posterior compartments are diploid and apoptose, whereas in the en-gal4 UAS-Rux disc posterior cells are iECs and resistant to cell death, while anterior diploid cells in that disc still apoptose.

... Finally, why is it that iEC don’t activate caspase? Is p53 never activated?..

**Response:** It is unknown why caspase is not activated in iECs after IR. We had previously shown that developmental endocycles inhibit apoptosis through chromatin silencing of p53 target genes, and not because of a failure of upstream activation of p53 (Zhang et al 2014, PLoS Genetics Vol. 10 Issue 9 Pages e1004581). We had also previously shown that iEC do not activate reporters of the p53 target gene *hid* after IR, suggesting the mechanism of apoptotic repression may be similar in induced endocycling cells (Hassel et al. 2014 Development Vol. 141 Issue 1 Pages 112-23). However, we have not defined the full mechanism by which apoptosis is repressed in iEC nor how this is linked to cell cycle program, both important questions for future studies.

4. The polytenization of the highly polyploid iEC after JNK inhibition is striking and very interesting. In addition to showing that JNK signaling is required for attenuation of tissue growth via senescence, it suggests that devEC don’t activate JNK; e.g. in the highly polyploid and polytene salivary glands. Is that known? What would happen if JNK was ectopically activated in the salivary gland? Would that block endoreplication and/or polytenization?

**Response:** We have addressed this question by expressing an activated Jun Kinase Kinase (*hep^CA^*), which severely inhibited endocycles and growth of salivary glands (Fig. S6A-D). We have no evidence that JNK affects polyteny directly, it is just more evident in the more highly polyploid cells. Polyteny is the default state for all polyploid cells in *Drosophila*, as evidenced by FISH labeling always resulting in one nuclear focus, even in those cells where polyteny is not evident by DAPI labeling.

5. Figure 6O-Q: it’s hard to tell what’s really going on here. Can the spindle be labeled to better demonstrate multipolarity? Are those PH3 positive chromosome fragments all in the same iEC? A cell cortex marker might help in this regard.

**Response:** We have extended this data which is now shown in a new Figure 7. We have labeled mitotic chromosomes with anti-PH3 and the cell cortex with anti-Fas3, which demonstrates that the polyploid mitoses are indeed in one cell (Fig. 7D-D'). Labeling with beta-tubulin also demonstrated that the mitotic spindle is multipolar (Fig. 7E-E'). We have also extended these experiments by examining later time points, and by lineage analysis, which shows that some iEC daughter cells can continue to proliferate (Fig. 7J-L''').

6. The experiments in Figure 7 are the least well developed and thus the least convincing. In panel B there appears not to be EdU labeling in the anterior half of the disc, unlike control. Why is that? That’s not true for other discs shown in the manuscript. Also, the data are not quantified. In addition, why would you expect to regenerate a “normal wing” if there's already a bunch of polyploid iEC in the posterior compartment? The logic for this expectation doesn’t make sense. This line of experimentation seems a bit preliminary and could be removed.

**Response:** We have chosen to retain and quantify the observations on compensatory proliferation because we believe that they have important developmental and biomedical implications (now Figure 8). This wing disc looks different because it is a later time point after endocycle induction (96 hours) then most other discs shown in the paper (although see Fig 3 H, I and Fig 4 D-D" for similar 96 hour disc images). It is during this later time point that the compensatory proliferation of diploid cells near the undergrown iEC is most evident. We have now quantified EdU labeling which shows increased division of diploid cells near the arrested iEC (Figure 8C-D, E-F'). With regard to reduced EdU labeling in the rest of the disc, this is a known phenomenon of the disc wounding / regeneration response, during which activation of DILP-8 inhibits wing disc cell cycles distant from the wound site. We have added a sentence to results to clarify this point (page 15, para first). With regard to regeneration of a normal wing, classic experiments of the 20th century showed that wing discs have an amazing ability to regenerate even after large regions are surgically removed. More modern approaches to kill or create loser cells have shown that those cells are eliminated from the disc epithelium and compensatory proliferation of the remaining cells can regenerate a normal wing disc and adult wing. We therefore thought it appropriate to determine whether a similar response occurs after iEC undergrowth. We found that compensatory proliferation does occur, but fails to regenerate a normal disc. As we discuss, the fact that iEC do not die and do not delaminate from the epithelium is interesting because it makes them different from other growth-compromised cells, with important broader implications for development and cancer.

**Reviewer #3:**

1. Fig. 6O-Q’ is confusing. Based on the mitotic marker PH3, endocycling cells can return to a mitotic state once rux has been shut off, leading the authors to conclude that senescence in iECs is reversible. However, senescence marker SA-beta-gal is, if anything, higher after rux shut off. So, based on this marker, iEC cells are still in senescence. If the senescence is really reversible, shouldn’t SA-beta-gal also disappear? If it persists because it is a stable protein, the authors should monitor other senescence markers with shorter half-lives to test their idea more rigorously.

**Response:** We have extended this analysis in a new Figure 7. The main point of this figure is that iEC senescent arrest is reversible. We have now conducted a lineage analysis that shows that at a least a subset of iEC daughter cells can continue to divide (Fig. 7F-L'''). Before we embarked on this analysis, we had hoped that SA-beta-gal activity would perdure into the first mitosis to rigorously prove that the cell had previously been in a senescent arrest, essentially the motivation for the PH3 / SA-beta-gal double labeling. The reversibility of senescence is also consistent with the observation that 100% of cells in the wing pouch polyploidize and that we never see PH3 labeling with constant Rux expression. In the current manuscript, we have further evaluated SA-beta-gal activity in longer time points after return to division (3 days), which showed that SA-beta-gal activity is extremely diminished but not completely absent in some cells (Fig. 7F-I'). At present we cannot determine whether this is just perdurance of SA-beta-gal protein or a continued low level of transcription. While SA-beta-gal is a widely used marker for senescence in flies and mammals, it remains unclear what its function is in senescent cells and what constitutes the best marker for what is now known to be a diversity of senescent states (Valieva et al. Diagnostics (Basel) 2022 Vol. 12 Issue 10). Whether cells that return to division retain some senescent gene expression or other senescent qualities is an interesting question that we believe is beyond the scope of this study.

2. Fig. 7 is also confusing and does not appear to show what the authors describe. We are supposed to see increased proliferation (EdU) of diploid cells that surround iEC cells, but the most striking results seems to be the reduction of EdU to near absence in the diploid cells of the anterior compartment. The RFP+ posterior compartment appears highly fragmented so are the ‘diploid neighbors’ posterior cells that somehow did not express rux? Or are they anterior cells that are now next to posterior cells? Overall, the possibility that iEC cells induce non-autonomous proliferation is better addressed using well-isolated iEC clones as in Fig. 1 where the boundary between iEC and diploid neighbors are clearly defined.

**Response:** Both you and reviewer #2 had questions about the observations that iEC can induce proliferation of diploid neighbors. We have extended this analysis, which further supports that iEC undergrowth induces proliferation of diploid neighboring cells (now Figure 8). We have quantified EdU labeling which shows increased division of diploid cells near the arrested iEC (Figure 8C-D, E-F'). With regard to reduced EdU labeling in the rest of the disc, this is a known phenomenon of the disc wounding / regeneration response. It was shown that activation of DILP-8 during wounding / regeneration inhibits wing disc cell cycles distant from the wound site, consistent with what we observe. We have added a sentence to results to clarify this point (page 15, para first). To evaluate the identity of cells, we have marked anterior cells with anti-Ci and posterior cells with enGAL4>RFP expression (Fig 8E-F"). This showed that iEC retain posterior fate and are surrounded by proliferating Ci-positive anterior cells, consistent with previous observations that compartment lineage boundaries are not obeyed during severe wounding and regeneration.

This is a minor concern but the effect of p35 co-expression may be better tested in the background where rux expression results in a greater reduction in growth than what is shown in Fig. 3F (for example, the 96 hr condition in Fig. 2C).

**Response:** We have quantified the effect of p35 expression at the 96 hr time point, which showed that it does not rescue iEC tissue undergrowth, further supporting that apoptosis does not make a major contribution to disc undergrowth (Fig 3G-J).

For improved presentation, please label the panels with the name of GAL4 driver in Fig. 2 (en or pdm2). And clarify whether the published RNAseq dataset from S2 cells induced to endocycle (re-analyzed in supplemental Table 1) are from cells induced with cyclin A dsRNA or Myb dsRNA (since both were used to induce endocycles in the prior publication).

**Response:** We have labeled Fig 2 with GAL4 drivers as requested. We have added text to results and methods to clarify that senescent gene list is from CycA RNAi (page 11, para 1 and page 20, para last).
